# Rethinking Assessment Concepts in Dental Education

**DOI:** 10.1155/2020/8672303

**Published:** 2020-10-14

**Authors:** Mohamed El-Kishawi, Khaled Khalaf, Dana Al-Najjar, Zahra Seraj, Sausan Al Kawas

**Affiliations:** Department of Preventive and Restorative Dentistry, University of Sharjah, PO Box 27272, Sharjah, UAE

## Abstract

**Introduction:**

Dental education involves teaching and assessing the acquisition of verifiable domains that require superior psychomotor, communication, and cognitive skills. Evolving technologies and methods of assessment could enhance student's learning environment and improve tutor assessment experience. The aim of this study was to review the current body of research and evaluate the effectiveness of various methods of assessments in improving learning and performance in preclinical and clinical dental practice.

**Materials and Methods:**

A search strategy was implemented using electronic search in major databases. The following key terms, clinical skills, preclinical, dental students, and assessment, were included in the search. Two reviewers independently screened all the articles retrieved following very specific inclusion criteria.

**Results:**

The initial search generated 5371 articles and 24 articles were selected for review and data extraction. Cohen's kappa coefficient was used to measure interrater agreement and a score of 94.7% was obtained.

**Conclusion:**

Preclinical assessment is an effective tool for promoting skills transfer to clinical phase. Early psychomotor skills assessment is valuable. It allows early intervention in the learning process and assists in effective utilization of learning resources. Technology-enhanced assessment systems allow better patient simulation, enhance learning and self-assessment experiences, and improve performance evaluation. However, these systems serve as an adjunct to conventional assessment methods. Further research should aim at calibrating and integrating these systems to optimize students learning and performance.

## 1. Introduction

Assessment is a crucial component in the learning process because most students focus on assessment more than any other component of their program; hence, it has the power to drive student learning [1, 2]. Preclinical and clinical dental education relies on suitable curriculum design in order to ensure that the desired learning outcomes meet with the level of competency required and enable dental graduates to practice autonomously. Additionally, assessment requirements define the curriculum for most students [3]. Therefore, assessment tasks can be used by educators to optimize the learning potential of students by ensuring that these assessments concentrate on critical thinking, self-directed, and lifelong learning and inspire innovation and creativity in their education [4].

Various assessment methods have been discussed within the body of literature in dental education; however, the selection of assessment method depends on the purpose of its use, whether it is for summative or formative or both. Summative assessment is outcome dependent while formative assessment relates to in-process evaluation of student's performance [5]. Different assessment tool criteria have been identified as attributes of an assessment method including interpretation and proper use of the results of assessment (validity), consistency of a test taker's score on an assessment when repeated on more than one occasion (reliability), learning opportunities delivered through a well-designed assessment, and feasibility of the assessment method (cost-effectiveness) [6]. Nevertheless, a single assessment method will not assess all domains of student's performance, and each method has its strengths and weaknesses. Thus, the use of various assessment methods is essential in order to compensate the shortcomings of one method by the advantages of another and the choice should be dictated by fitness for purpose [7].

The purpose of assessment of learning is to decide whether a student has successfully achieved a learning outcome. This type of assessment does not aid in the improvement of the learning process [5]. However, assessment for learning can help provide feedback to both educator and learner regarding the learner's progress towards meeting the objectives [8]. This feedback should be used by the educator to revise and develop further instruction. Feedback is used to actively improve student learning and may be informative and supportive and help facilitate a positive attitude towards future learning [2]. However, the limitations of conventional teaching methods have been reported and include lack of realism in simulation, limited tutor resources and time, and subjectivity of the assessment methods and feedback [9]. To overcome these limitations, the use of computerized dental assessment systems has been suggested as an effective and reliable tool [10].

Assessment methods in dental education are a broad topic, and upon a preliminary search, a large volume of studies investigating many aspects of assessment were found. Therefore, the aim of this review was to explore the current body of research and evaluate the effectiveness of various methods of assessments applied to predoctoral dental education in preclinical and clinical settings for the purpose of improving learning and performance in dental practice. This review sought to answer three research questions. (i) What was the scope of the studies that have been reported on preclinical and clinical dental assessments? (ii) What was the quality of research studies available? (iii) What were the main themes in the literature regarding preclinical and clinical assessment in dental education?

## 2. Materials and Methods

A search strategy was implemented using the following four databases: PubMed, ScienceDirect, Google Scholar, and Embase and the key terms that were applied were “clinical skills,” “preclinical,” “dental students,” and “assessment.” Additionally, the Journal of Dental Education was manually searched to identify possible studies that may not be indexed in major electronic databases.

Inclusion and exclusion criteria were set according to the Population, Intervention, Comparator, and Outcome (PICO) model [11]. The study population was limited to predoctoral dental students in a preclinical and clinical setting. The intervention was preclinical/clinical assessment of daily procedures in both formative and/or summative format. The comparator was conventional faculty assessment; however, this was not an excluding factor if absent. The outcome was agreement level between the different assessment methods. Peer-reviewed articles written in English published in the period between Jan 2009 and Jan 2020 were included as well as both quantitative and qualitative studies, whereby the preclinical/clinical assessment tool was the main focus/intervention. Studies were excluded if written in languages other than English, the sample was not solely predoctoral dental students and the preclinical/clinical assessment tool was not the main focus, publications targeted a sample of postgraduate dental students, and/or anything published before January 2009.

Two reviewers independently screened all the articles generated by both manual and electronic searches. The screening and inclusion processes were managed in three phases: initial identification of articles, determining eligibility for inclusion, and selection of articles for review. Cohen's kappa coefficient was used to measure interrater agreement, and a score of 94.7% was obtained.

## 3. Results

**Figure 1:**
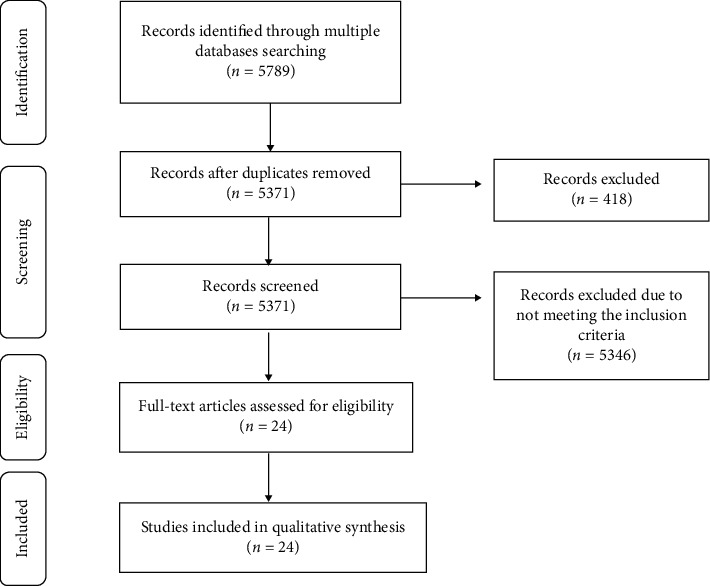
Flow diagram of the process of study selection.

Figure 1 shows the process of study selection that resulted in the final 24 studies for inclusion in the final analysis.

The search strategy generated 5789 articles from which a total of 418 duplicates were removed. 5371 articles were screened according to the titles and abstracts and 5347 articles were excluded as they did not meet the inclusion criteria. The full texts of the remaining 24 articles were retrieved for data extraction and assessment by two co-authors independently (Figure 1). These studies are summarized in Table 1.

Two authors reviewed the potential risk of bias. The assessment was achieved independently and in duplicate. The Cochrane risk of bias tool was used as an assessment tool [11]. The risk of bias was weighed and evaluated for domains which included random sequence generation, allocation, concealment, blinding of participants and personnel, blinding of outcome assessment, incomplete outcome data, and selective outcome reporting. For each of the domains, studies were judged for having risk of bias as high, low, or unclear.

The overall risk of bias was assigned according to the following categories:

  Low risk: all main domains were at low risk of bias
  High risk: one or more main domains of the study were at high risk of bias
  Unclear risk: one or more main domains of the study were unclear

This systematic process resulted in 24 articles being selected for inclusion in the review, seven had a low risk of bias [10, 14, 15, 21, 22, 24, 27], six had a high risk of bias [12, 16, 20, 29, 30, 32], and eleven had an unclear risk of bias [13, 17–19, 23, 25, 26, 28, 31, 33, 34].

These 24 articles were thoroughly reviewed for the key outcome measures: sample size, student year in program, clinical or preclinical assessment, faculty assessment, faculty calibration, grading rubric, and use of the same form in the preclinical and clinical environments. The complete listings of articles and data generated from each included study are shown in Tables 1 and 2. Cohen's kappa coefficient was used to measure interrater agreement and a score of 94.7% was obtained.

## 4. Discussion

Assessment for learning is an educational concept that motivates both the educator and learner to actively improve the learning process and facilitate a positive attitude towards future learning. Assessment in dental education should include a diagnostic component in order to identify learning barriers and student weaknesses [35, 36]. The assessment of various domains of competence in dental education requires multiple methods of assessments and constructive feedback in order to overcome the limitations of single assessment formats. Dental educators should be aware of the limitations of each method of assessment and its impact on learning [37]. The Commission on Dental Accreditation (CODA) standards state that “graduates must demonstrate the ability to self-assess, including the development of professional competencies and the demonstration of professional values and capacities associated with self-directed, lifelong learning” [38].

This review involved studies that addressed preclinical and clinical assessment in dental education. Most of the studies presented their methodology with a faculty calibration system and a clear grading rubric related to the assessment method used. However, many of these studies had an unclear risk of bias, which was related to their random sequence allocation and allocation concealment. The discussion regarding these studies was arranged according to the three themes identified among the literature: self-assessment, preclinical assessment as an indicator for clinical performance, and technology enhanced assessment.

### 4.1. Self-Assessment

Self-assessment is a useful tool in dental education, which is utilized in many dental colleges following exercises performed in preclinical or clinical practice. It can be used in both formative and summative activities. It is a method used for dental students to improve self-directed learning, lifelong learning, and cognitive abilities [39]. However, it has been reported that students find it challenging to self-assess, and therefore, it requires adequate training [17, 29]. Investigators reported that students had challenges in self-assessment on cavity preparation tasks and the amount of student-faculty agreement was not improved through digital assessment [29].

It was shown that performance assessment in preclinical operative dentistry was overestimated by students compared to faculty assessment [17]. A strong correlation was found between preclinical performance and self-assessment accuracy whereby students with low performance significantly overestimated their assessments, and self-assessments of higher performing students were much more accurate [17]. Thus, regular feedback during formative assessment can gradually develop and improve student's ability for accurate self-assessment, which can be further evident during their summative assessment. Students' self-assessment ability improved when given the opportunity to reflect on their feedback from multiple sources and apply critical thinking in the process of producing their self-generated study plan [20]. Therefore, self-assessment should start early in preclinical stages, using a similar structure of self-assessment in the clinical environment to increase its predictive value [29, 40].

### 4.2. Preclinical Assessment as an Indicator for Clinical Performance

Preclinical courses are designed to enable students to achieve high standard patient-care in dental clinical practice. The use of conventional typodont (mannequin head) has been always considered a valuable tool for simulating patient care procedures [32]. However, it is of more value when teaching basic principles such as learning ergonomic positions, familiarity with the instruments, and performing simple tasks such as cavity and crown preparations in a safe environment and standardised manner, but it is not useful for patient care simulation as the context is not real, because every patient is different in terms of mouth opening, dynamic occlusion, saliva, pain, and cooperation. Two studies measured the association between students' performance in preclinical (on mannequin head) and clinical courses (on actual patient) [32, 41]. No correlation in outcomes between mannequin head and actual patient was detected. Additionally, mannequin-head simulation was not a suitable measure of competency and clinical skills. However, another study reported a positive link between preclinical and clinical performance. Thus, preclinical performance may have a weak predictive effect on clinical performance, indicating the presence of further contributing factors [28]. The use of computerized dental assessment was proposed to overcome the limitations of using typodont in preclinical dental teaching. It enables immediate feedback during clinical training that is suggested to improve student performance during preclinical simulations and may be used as an initial predictor for clinical performance [10, 33].

Practical grades represent an overall evaluation of a broad range of student's preparation performance. Students tend to make errors in pattern preparation exercises, width, depth, or both, in the preclinical practical stage. Early intervention may enable faster development of hand-piece manipulation skills, as assessed by performance on practical work provided early in the course [34]. However, this study did not indicate whether early identification and remediation of perceptual or technical errors may result in subsequent practical performance at a desirable level. Prediction of clinical performance at a clinical stage is complex and multifactorial; it relies on the consistency of cognitive and psychomotor skill improvement through the three phases of skill acquisition: the cognitive phase, associative phase, and autonomous phase [42, 43].

Objective structured clinical examination (OSCE) is considered one of the common assessment methods applied in clinical dentistry. It is meant to assess clinical competencies in history taking, clinical examination, interpretation of laboratory and radiographic findings/special investigations, mastery of procedural skills, counselling, attitudinal behavior, and communication skills. It should consist of adequate number of stations (typically 10–15 stations) through which students have to rotate from one station to the next at the signal. A student's performance in a station is scored against an agreed-upon checklist or rating scale. An investigation of the relationship between student performance on OSCE and clinical performance showed a positive correlation as based on clinical productivity [30]. The administration of a comprehensive, multidisciplinary OSCE prior to clinical learning stage might be an effective and reliable indicative educational method in dental education [30]. Further research is needed regarding this topic as we only identified one article investigating the relationship between OSCE and clinical performance within the period of this review.

### 4.3. Technology-Enhanced Assessment

Computerized dental simulator (CDS) and virtual self-assessment (VSA) can offer a potential alternative feedback and assessment tools, which can enhance student's learning and self-assessment experiences [10, 24]. CDS methods can provide a valuable perspective that is not offered by conventional assessments. It allows for the manipulation of the 3D model by means of rotating and zooming, which simulates everyday technology and games, as clinical work can be viewed in different angles. It can also provide automated and immediate assessment [12, 27].

CDS along with the conventional typodont simulation systems provide enhanced spatial awareness and fine-motor coordination and enrich the depth and auditory perception. CDS can enhance psychomotor skill performance and improve the assessment accuracy of these skills [16]. Using psychometric tests can help demonstrate the growth of specific sets of skills amongst students that may not be recognized through traditional assessment methods. CDS was tested for its ability to predict preclinical performance in operative dentistry and prosthodontics [10, 33]. This includes the use of simulation exam scores, frequency of student self-assessment, accuracy, and completion time of both simulation task and self-evaluations [19]. It was found that early use of CDS can assist in recognizing students in need for early instructional modification. This highlighted the importance of early psychomotor skills assessment in dentistry. It was found that dental students' learning process may benefit from CDS scores, which in turn allows early intervention and better distribution of learning and teaching resources, including the number and placement of teaching staff [19].

VSA software can provide a significant learning opportunity that enables students to accurately and effectively carry out self-assessment [13]. The assessment ability of deficient students benefitted the most, matching their ability to the standard achieved by the rest of the class. When conventional assessment was compared to VSA, it was found that hand-eye grading was limited by poor interrater reliability and low interrater consistency. VSA demonstrated potential as an evaluation process as it had excellent interrater reliability and correlation [23]. The use of VSA software to assess student's skills was found to provide feedback that was both reliable and repeatable [15, 22, 25]. E4D Compare [10, 15, 18, 22, 24, 31], prepCheck [21, 22], Computer Aided Design, Computer Aided Manufacturing (CAD CAM) [27, 29], Cavity Preparation Skill Evaluation System (CPSES) [23], CEREC Omnicam [13], and DentSim [33] were among the commonly reported VSA systems in the literature. Evaluations provided by these systems were significantly more consistently accurate for some aspects of the dental tasks (e.g., surface reduction, wall height, and undercuts) than conventional-grading by teaching staff [25]. However, further research is warranted to adjust the software's parameters related to acceptable variations from the ideal situation (tolerance level) [24]. It was also recommended to further assess the reliability of these systems as a self-assessment method and as a tutor calibration tool in dental education [25].

E4D Compare scanning software was reported as a reliable VSA tool for matching and comparing standard ideal tooth preparation with preparations produced by students [31]. Additionally, it was able to match the efficacy of clinical staff in providing instant feedback on tooth preparation [14]. However, the software was unable to consistently identify some common critical assessment criteria, such as occlusal clearance and configurations of finish lines [12]. Therefore, it was recommended that further research into the VSA software is needed, in order to determine acceptable tolerance levels among educators and to use a variety of methods in calibrating clinical staff, intra-assessor reliability, and accuracy [12, 27]. Studies suggested that further evaluation of the proper application of these computer-generated numbers and recommended the development of an appropriate grading model (rubric) that uses VSA technology [26, 27]. These programs should ideally be structured to grade all aspects of the dental task, making it as objective as possible [18].

Despite the advantages of the CDS and VSA resources, these resources can have some deficiencies including the need for preusage training and calibration, modification of conventional nonstandard teaching methods, evaluation restrictions, licensing expenses, maintenance, and other consumables costs involved. These methods of assessment can serve in shaping the learning experience and facilitate an autonomous transfer of the acquired clinical skills. This may be very effective as an adjunctive educational tool, allowing students to accurately and critically evaluate and compare their work and recognize their deficiencies, especially among the lower performing students. However, digital assessment should not replace conventional assessments; rather it should be integrated into the dental curriculum to supplement conventional assessment and to improve assessment ability [21].

Our findings showed that CDS and VSA resources allow students to improve their learning and performance by potentially enhancing their learning curve and giving them the chance to evaluate their learning outcomes in a simulated setting prior to skill application in real patient care settings [34]. Thus, implementation of enhanced information technology could be used to compensate for the lack of clinical teaching staff. Moreover, a supplementation with this digital aid may provide the opportunity to focus on assisting lower-performing students for better development of their psychomotor skills during patient simulated tasks [10].

Regardless of the assessment method used, dental educators need to make sure that they are evaluating their learner's acquisition of knowledge, skills, behaviors, and professional qualities and expertise for safe and competent dental practice. In the future, performance-based assessment will dominate in dental education, where students will be assessed as a member of a multiprofessional team tackling together a series of complex scenarios encountered during clinical practice. Future research must aim at developing a computerized dental assessment system that can accurately simulate dental tasks, gives early indication of students in need for psychomotor assistance, and can provide a learning tool that is based on contemporary educational concept allowing for a safe and feasible learning environment. Further studies should also focus on how and when to fit these systems in the dental curriculum along with the conventional teaching methods.

## 5. Conclusion

Preclinical assessment is an effective tool for promoting skills transfer to clinical phase; however, the correlation between outcomes of preclinical and clinical assessments is feeble. Early psychomotor skills assessment is valuable. It allows early intervention in the learning process and assists in effective utilization of learning resources. Technology enhanced assessment systems allow better patient simulation, enhance learning and self-assessment experiences, and improve performance evaluation. However, these systems can be used as an adjunct to complement deficiencies in conventional assessment methods. Further research is required to calibrate and integrate these systems to serve in optimizing students learning and performance. Future research must also aim at developing assessment system that accurately simulates dental tasks, gives early indication of students in need for psychomotor assistance, and can provide a learning tool that is based on contemporary educational concept allowing for a safe and feasible learning environment.

## Figures and Tables

**Table 1:** Summary of the studies included in the review.

Author, date	Sample size	Students	Preclinical assessment	Clinical assessment	Correlated faculty assessment
Furness 2018 et al. [12]	1	N/A	Planmeca compare software (crown preparations)	N/A	Yes
Lee 2018 et al. [13]	69	Third year	Self-assessment and faculty assessment; conventional vs. digital software (operative)	N/A	Yes
Sadid-Zadeh 2018 et al. [10]	9	Second year	Compare software (crown preparation)	N/A	Yes
Sadid-Zadeh 2018 et al. [14]	9	Second year	Compare software (crown preparations)	N/A	Yes
Sadid-zadeh 2018 et al. [15]	505	Second year	Compare software (crown preparations)	N/A	Yes
Shahriari-Rad 2017 et al. [16]	140	First/second year	Virtual haptic simulator (operative)	N/A	Yes
Lee 2017 et al. [17]	71	Third year	Self-assessment (operative)	N/A	Yes
Sly 2017 et al. [18]	98	First year	Compare software (operative)	N/A	Yes
Gottlieb 2017 et al. [19]	282	First/second year	Advanced simulation training	N/A	Yes
De Peralta 2017 et al. [20]	104	First year	Multisource assessment (operative)	N/A	Yes
Gratton 2017 et al. [21]	79	Second year	Compare and prepCheck software (crown preparation)	N/A	Yes
Gratton 2016 et al. [22]	80	Second year	E4D compare and sirona prepCheck software (crown preparation)	N/A	Yes
Zou 2016 et al. [23]	38	First year	Computerized cavity preparation evaluation system (operative)	N/A	Yes
Garrett 2015 et al. [24]	57	First year	Digital evaluation tool (dental anatomy wax-up)	N/A	Yes
McPherson 2015 et al. [25]	66	Not mentioned	Self-assessment software	N/A	Yes
Callan 2015 et al. [26]	10	Not mentioned	E4D compare software (crown preparation)	N/A	Yes
Callan 2014 et al. [27]	6 methods	N/A	CAD CAM assessment software (crown preparation)	N/A	N/A
Velayo 2014 et al. [28]	301	First to fourth year (cohort)	Preclinical performance	Preclinical performance as an indicator (operative and fixed prosthodontics)	Not mentioned
Mays 2014 and Levine [29]	25	First year	Using CAD CAM for self-assessment (operative)	N/A	Yes
Graham 2013 et al. [30]	145	Not mentioned	Comprehensive preclinical OSCE	Preclinical OSCE as a predictor for clinical performance.	Yes
Renne 2013 et al. [31]	50	Second year	E4D compare software (crown preparation)	N/A	Yes
Nunez 2012 et al. [32]	86	Fourth year	Preclinical typodont score as a predictor for clinical performance (fixed prosthodontics).	Preclinical typodont score as a predictor for clinical performance (fixed prosthodontics).	Yes
Urbankova and and Engebretson 2011 [33]	38	First year	Computer-assisted dental simulation (operative)	N/A	No
Boushell 2011 et al. [34]	81	First year	Learn-A-Prep II (operative)	N/A	Yes

**Table 2:** Assessment process of the studies included in the review.

Author, date	Assessment training	Predictive value	Faculty calibration	Grading rubric	Risk of bias
Furness 2018 et al. [12]	N/A	Software was not successful in identifying consistently common critical errors.	N/A	Yes, a well-defined grading rubric with criteria.	High
Lee 2018 et al. [13]	Yes	Lower performing students benefitted the most in improving their ability to self-assess.	Yes	Yes, a well-defined grading rubric with criteria.	Unclear
Sadid-zadeh 2018 et al. [10]	Yes	Compare software can be used to evaluate complete coverage crown preparations as interrater agreement between virtual software and faculty was almost perfect.	Yes	Yes, a well-defined grading rubric with criteria.	Low
Sadid-zadeh 2018 [14]	Yes	Compare software can be as effective in providing immediate feedback as faculty feedback.	Yes	Yes, a well-defined grading rubric with criteria.	Low
Sadid-zadeh 2018 et al. [15]	Yes	Compare software can be as effective in providing immediate feedback as faculty feedback.	Yes	Yes, a well-defined grading rubric with criteria.	Low
Shahriari-Rad 2017 et al. [16]	Yes	Haptic virtual reality software in combination with traditional phantom head mannequin is very effective in developing and assessing psychomotor skills.	N/A	Yes, a well-defined grading rubric with criteria.	High
Lee 2017 et al. [17]	Not mentioned	Low performing students overestimated their self-assessment and vice versa.	Yes	Yes, a well-defined grading rubric with criteria.	Unclear
Sly 2017 et al. [18]	Yes	Compare software was not comprehensive in grading intracoronal preparations.	Yes	Yes, a well-defined grading rubric with criteria.	Unclear
Gottlieb 2017 et al. [19]	Yes	Advanced simulator exam scores can be used as performance predictors in preclinical operative and fixed prosthodontics.	Not mentioned	Yes, a well-defined grading rubric with criteria.	Unclear
De Peralta 2017 et al. [20]	Yes	Use of multisource assessment improved student's ability to self-assess and interrater agreement with faculty.	Yes	Yes, a well-defined grading rubric with criteria.	High
Gratton 2017 et al. [21]	Yes	There was no significant difference between the use of compare software vs. prepcheck in students' performance.	Yes	Yes, a well-defined grading rubric with criteria.	Low
Gratton 2016 et al. [22]	Yes	Use of evaluation software had no effect on student's prosthodontics technical and self-evaluation abilities.	No	Yes, a well-defined grading rubric with criteria.	Low
Zou 2016 et al. [23]	Yes	Computerized cavity preparation evaluation system was a valuable tool for self-learning.	Yes	Yes, a well-defined grading rubric with criteria.	Unclear
Garrett 2015 et al. [24]	Yes	Conventional self-reflection and faculty guidance in conjunction with a digital evaluation tool can be used to teach students on how to perform self-assessments.	Yes	Yes, a well-defined grading rubric with criteria.	Low
McPherson 2015 et al. [25]	Yes	Software can be used for self-assessment and grading by faculty.	Yes	Yes, but not clear.	Unclear
Callan 2015 et al. [26]	N/A	Interchangeability of typodonts of the same make and model do not affect the accuracy of assessment.	Not mentioned	Yes, a well-defined grading rubric with criteria.	Unclear
Callan 2014 et al. [27]	N/A	“Small dots diagonal” on the gingiva was the best option.	No	Yes, but not clear.	Low
Velayo 2014 et al. [28]	N/A	Positive significant correlation between student's preclinical and clinical performance.	Not mentioned	Yes, a well-defined grading rubric with criteria.	Unclear
Mays and Levine [29]	Yes	Using CAD CAM did not improve student's self-assessment ability and poor agreement with faculty assessment was observed.	Yes	Yes, a well-defined grading rubric with criteria.	High
Graham 2013 et al. [30]	N/A	Preclinical OSCE was a reliable predictor of clinical performance.	N/A	Yes, a well-defined grading rubric with criteria.	High
Renne 2013 et al. [31]	N/A	E4D compare software was a reliable assessment tool.	Yes	Yes, a well-defined grading rubric with criteria.	Unclear
Nunez 2012 et al. [32]	N/A	Preclinical performance on typodonts was a poor predictor of clinical performance on live patients.	Yes	Yes, a well-defined grading rubric with criteria.	High
Urbankova and Engebretson 2011 [33]	Yes	Computer-assisted dental simulation test can identify students needing early instructional intervention	Yes	Yes, but not clear.	Unclear
Boushell 2011 et al. [34]	No	Learn-A-Prep II can be a good tool to identify students that may need early instructional intervention.	Not mentioned	Yes, a well-defined grading rubric with criteria.	Unclear
